# Supplementary material for: Unveiling a New Selenocyanate as a Multitarget Candidate with Anticancer, Antileishmanial and Antibacterial Potential
Source: Molecules. 2022 Nov 2;27(21):7477. doi: 10.3390/molecules27217477 (PMC9656702; doi:10.3390/molecules27217477)
Supplement: Supplementary file 1 [file molecules-27-07477-s001.zip › molecules-1986139-supplementary.pdf]

Unveiling a new selenocyanate as a multitarget candidate with anticancer, antileishmanial and antibacterial potential

Sandra Ramos-Inza [1,2], Andreina Henriquez-Figuereo [1,3], Esther Moreno [1,2,3], Melibea Berzosa [3,4], Ignacio Encío [2,5], Daniel Plano [1,2,3,*] and Carmen Sanmartín [1,2,3,*]

[1] Department of Pharmaceutical Technology and Chemistry, University of Navarra, Irunlarrea 1, E-31008 Pamplona, Spain
[2] Instituto de Investigación Sanitaria de Navarra (IdiSNA), Irunlarrea 3, E-31008 Pamplona, Spain
[3] Institute of Tropical Health of the University of Navarra (ISTUN), University of Navarra, Irunlarrea 1, E-31008 Pamplona, Spain
[4] Department of Microbiology and Parasitology, University of Navarra, Irunlarrea 1, E-31008 Pamplona, Spain
[5] Department of Health Sciences, Public University of Navarra, Avda. Barañain s/n, E-31008 Pamplona, Spain

Table of Contents

Chemical characterization

^{1}H, ^{13}C and ^{77}Se NMR spectra

**1a**	....	**Figures S1-S3**
**1b**	....	**Figures S4-S6**
**1c**	....	**Figures S7-S9**
**2a**	....	**Figures S10-S12**
**2b**	....	**Figures S13-S15**
**2c**	....	**Figures S16-S18**

- **Chemical characterization – NMR spectra**

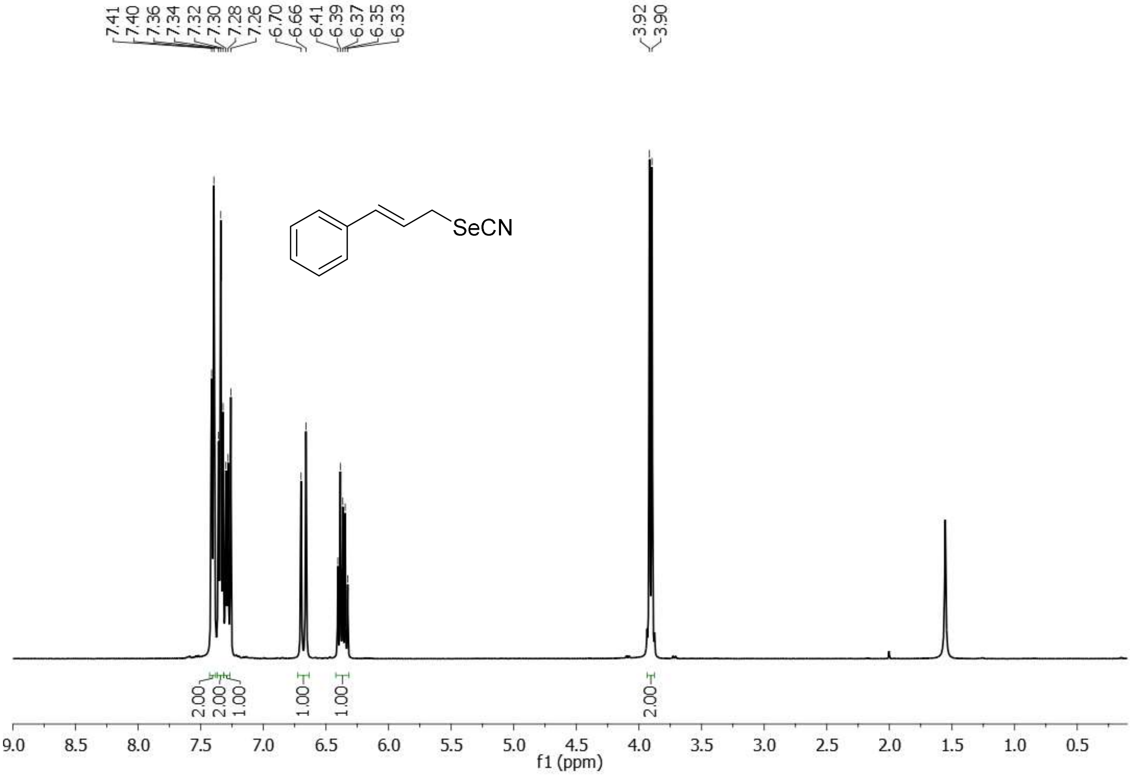

Figure S1. ^{1}H-NMR spectrum of compound **1a**.

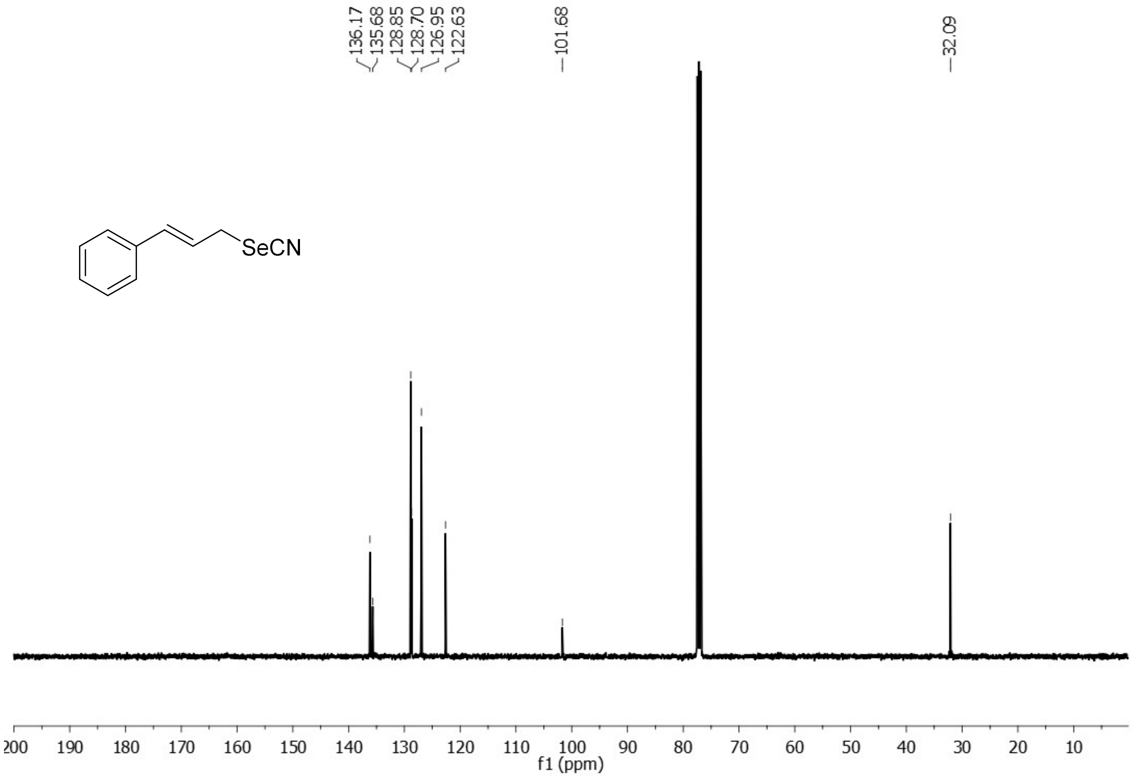

Figure S2. ^{13}C-NMR spectrum of compound **1a**.

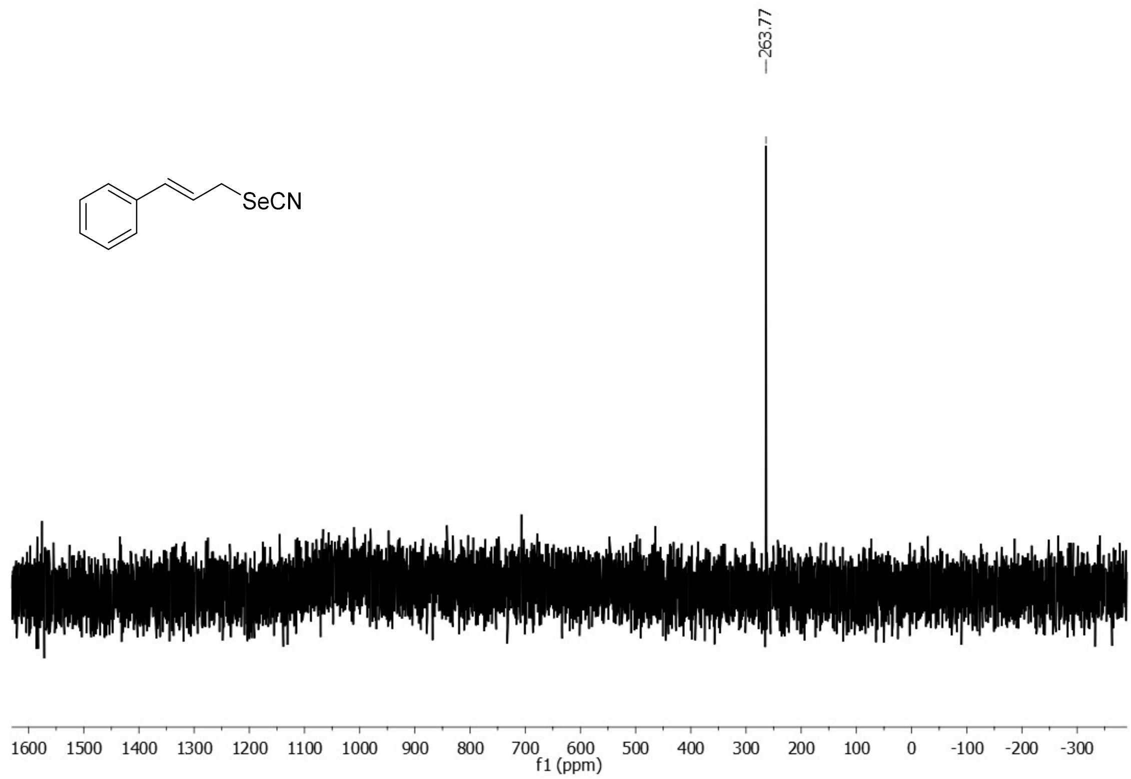

Figure S3. ^{77}Se-NMR spectrum of compound **1a**.

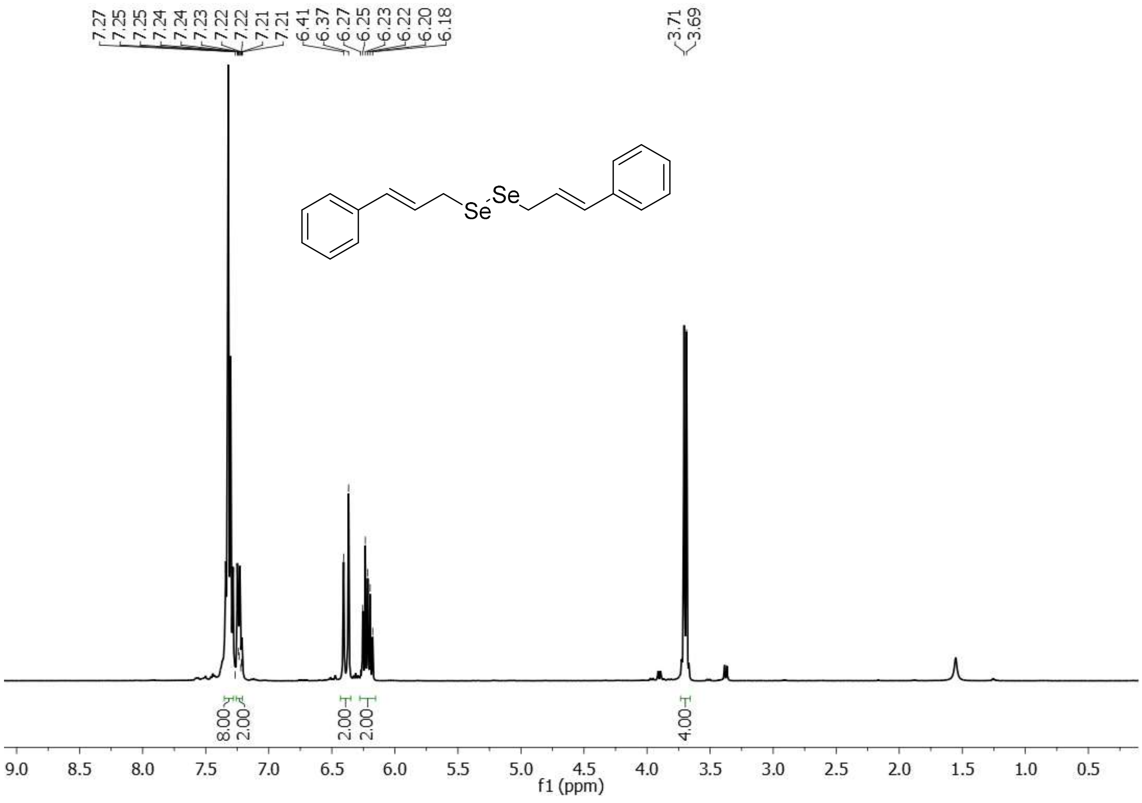

Figure S4. ^{1}H-NMR spectrum of compound **1b**.

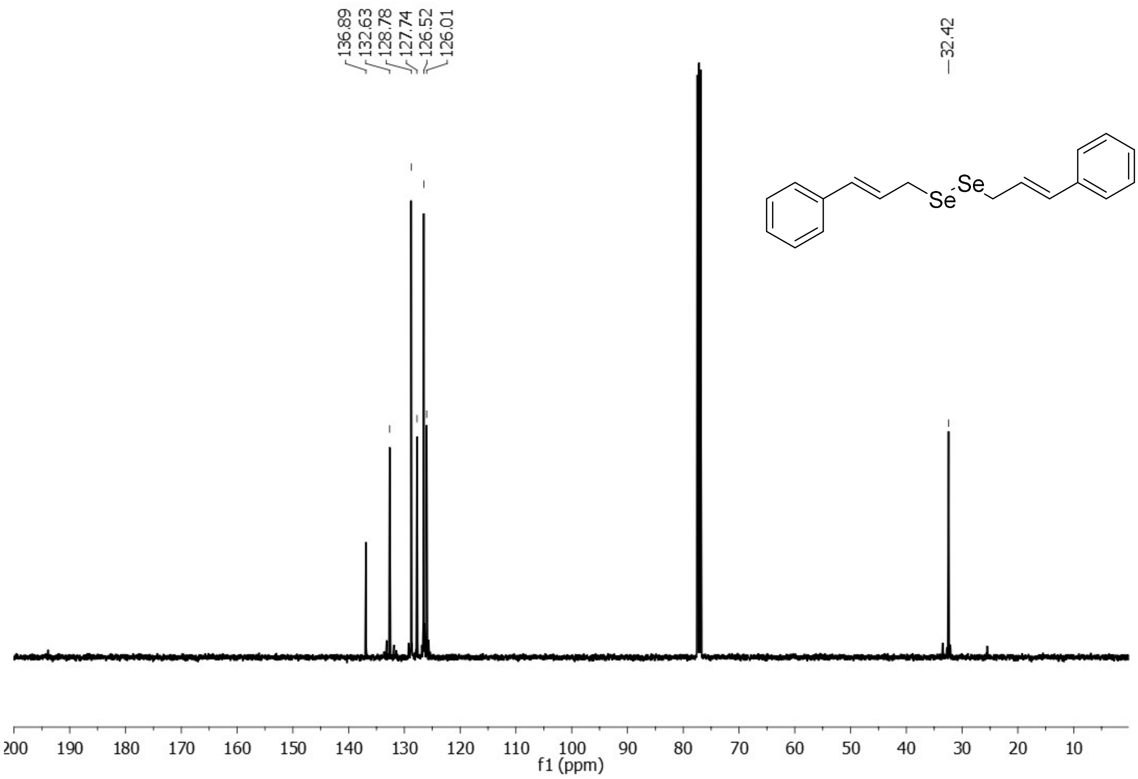

Figure S5. ^{13}C-NMR spectrum of compound **1b**.

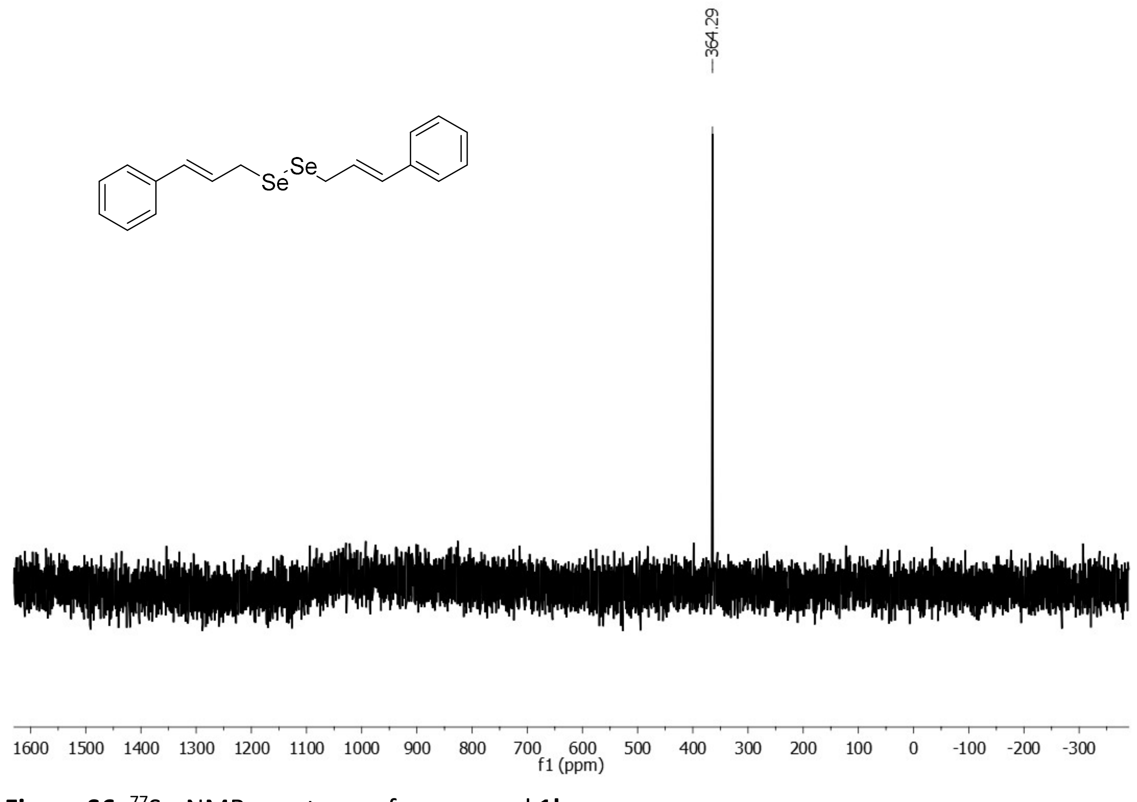

Figure S6. ^{77}Se-NMR spectrum of compound **1b**.

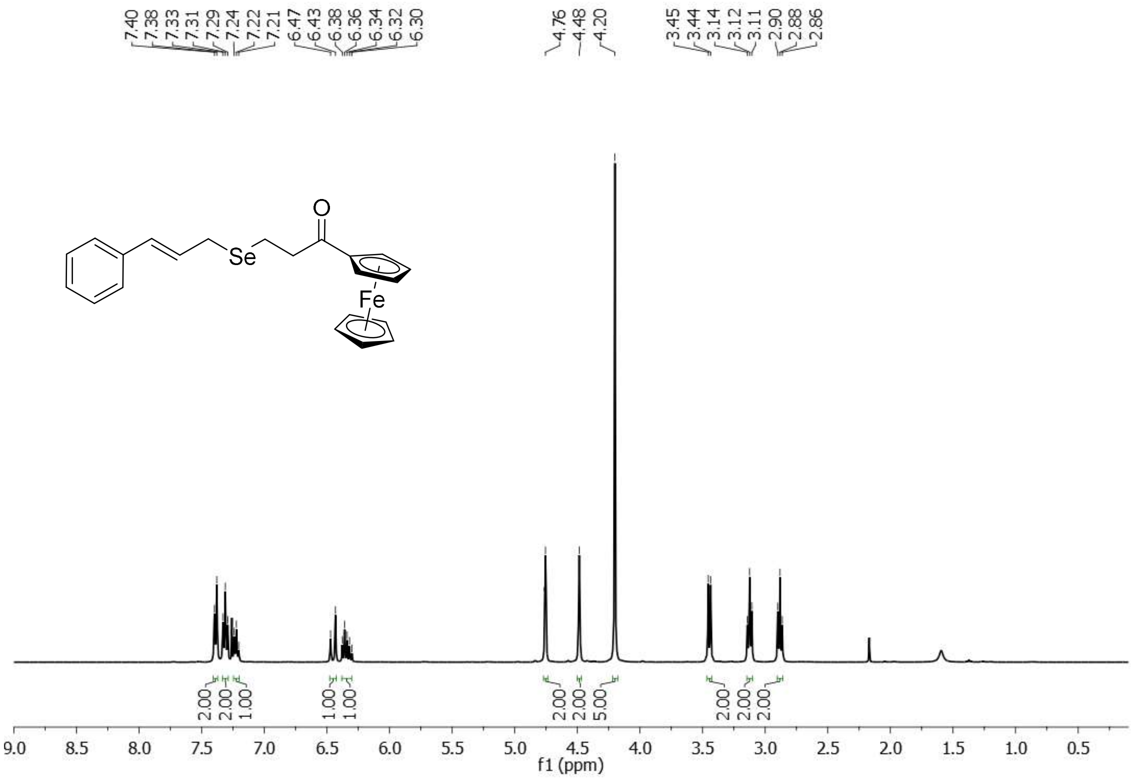

Figure S7. ^{1}H-NMR spectrum of compound **1c**.

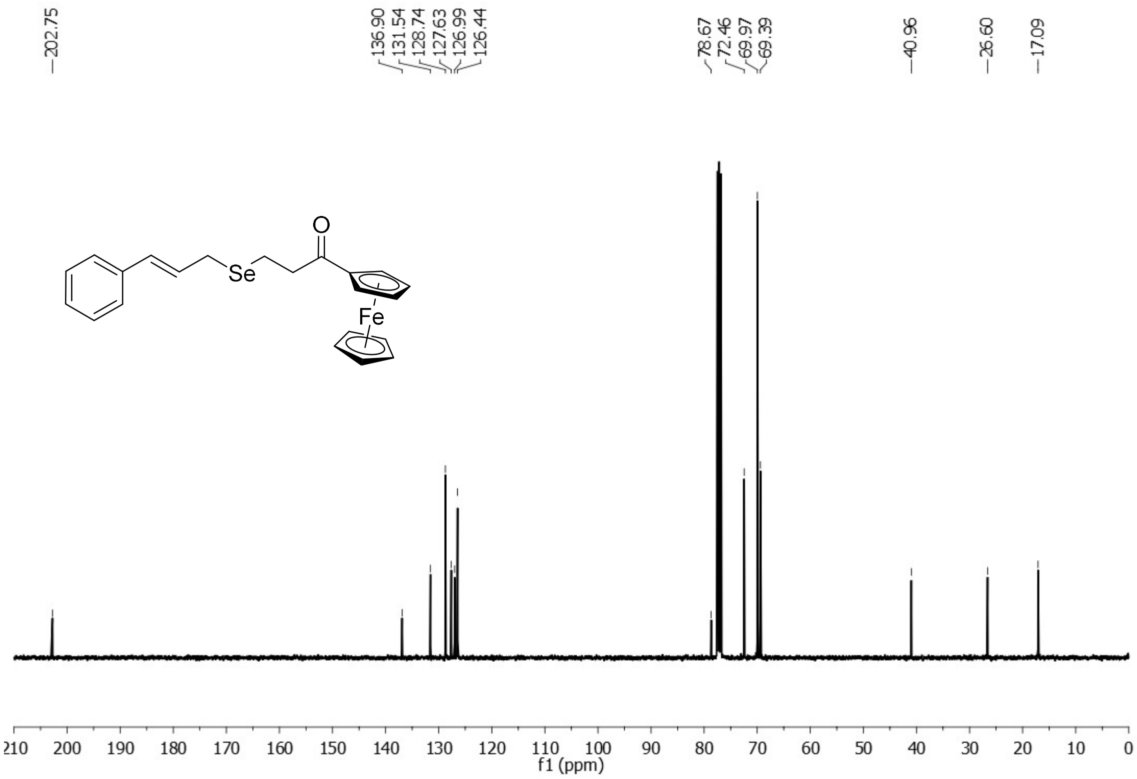

Figure S8. ^{13}C-NMR spectrum of compound **1c**.

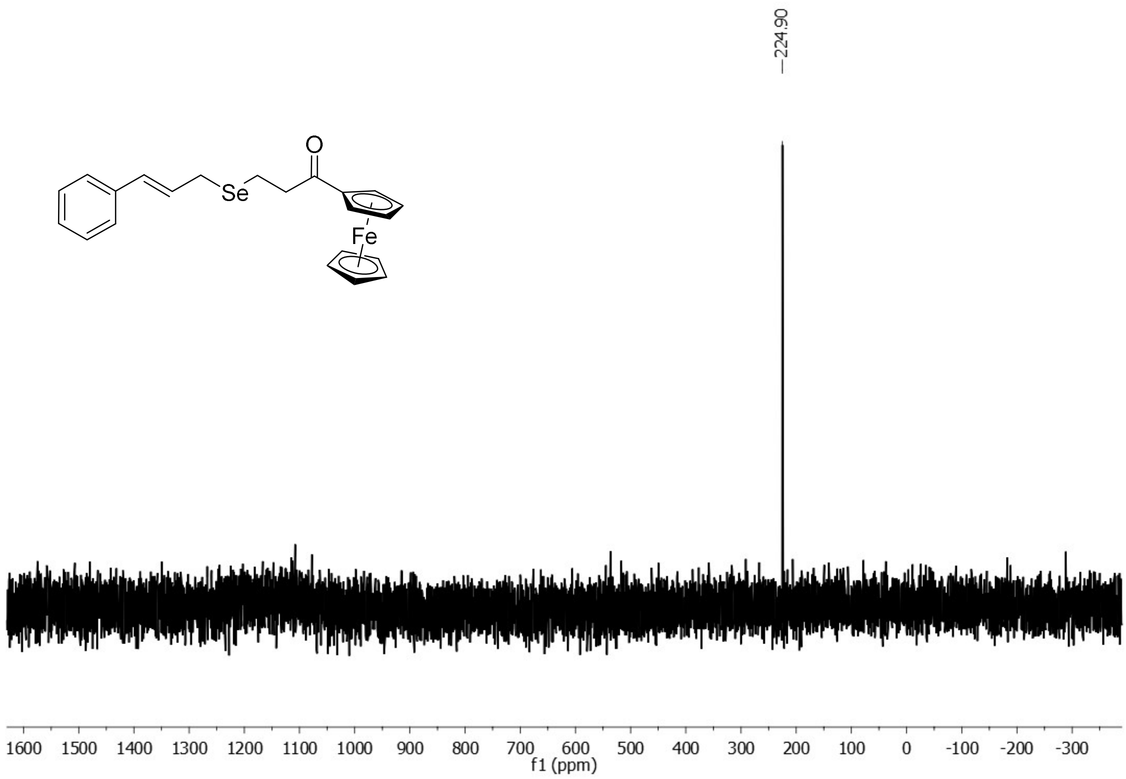

Figure S9. ^{77}Se-NMR spectrum of compound **1c**.

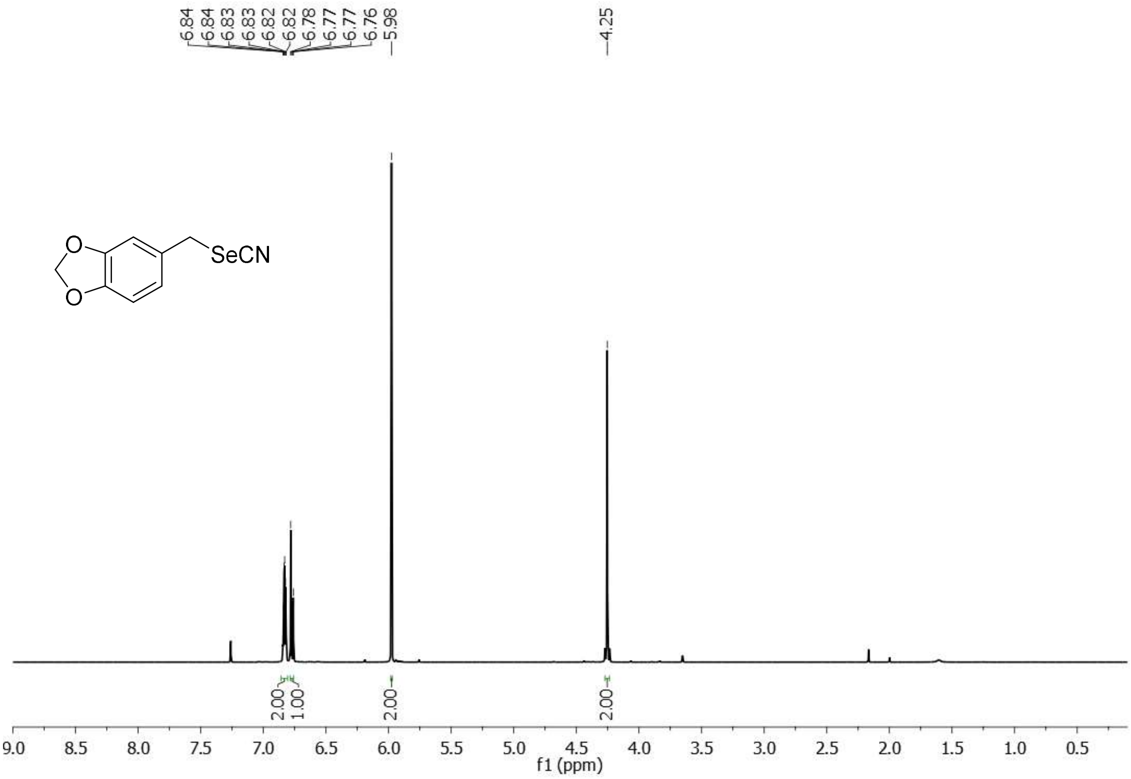

Figure S10. ^{1}H-NMR spectrum of compound **2a**.

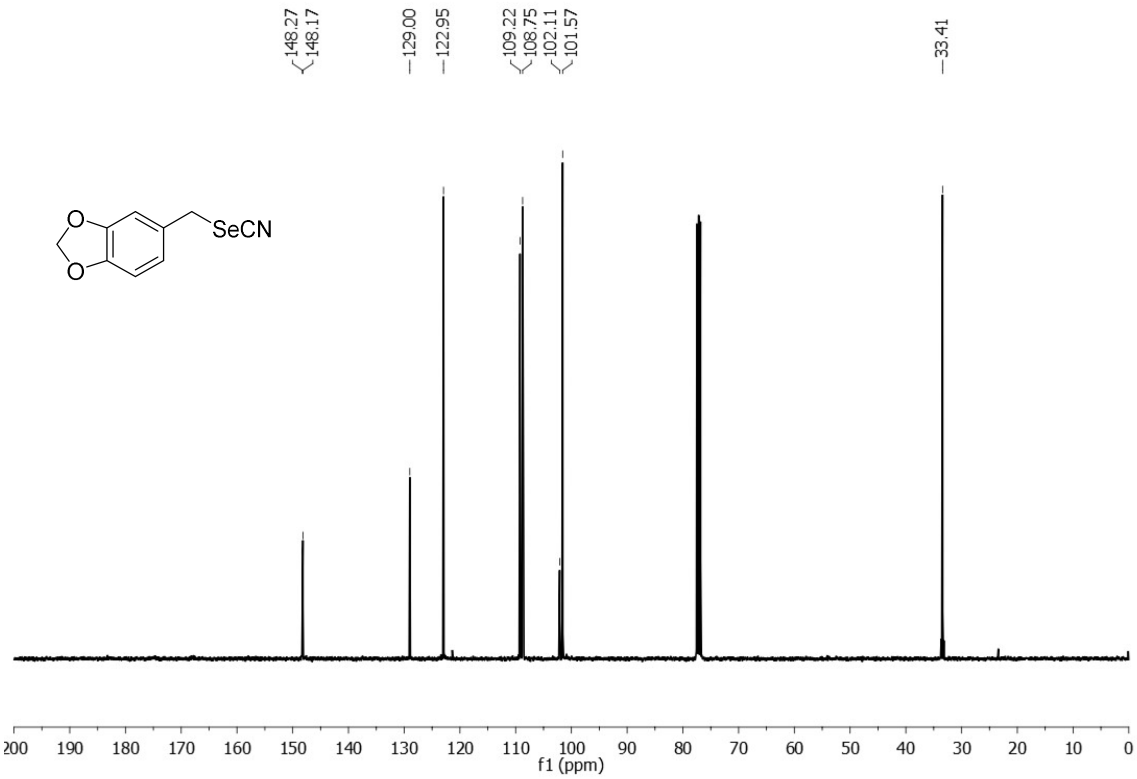

Figure S11. ^{13}C-NMR spectrum of compound **2a**.

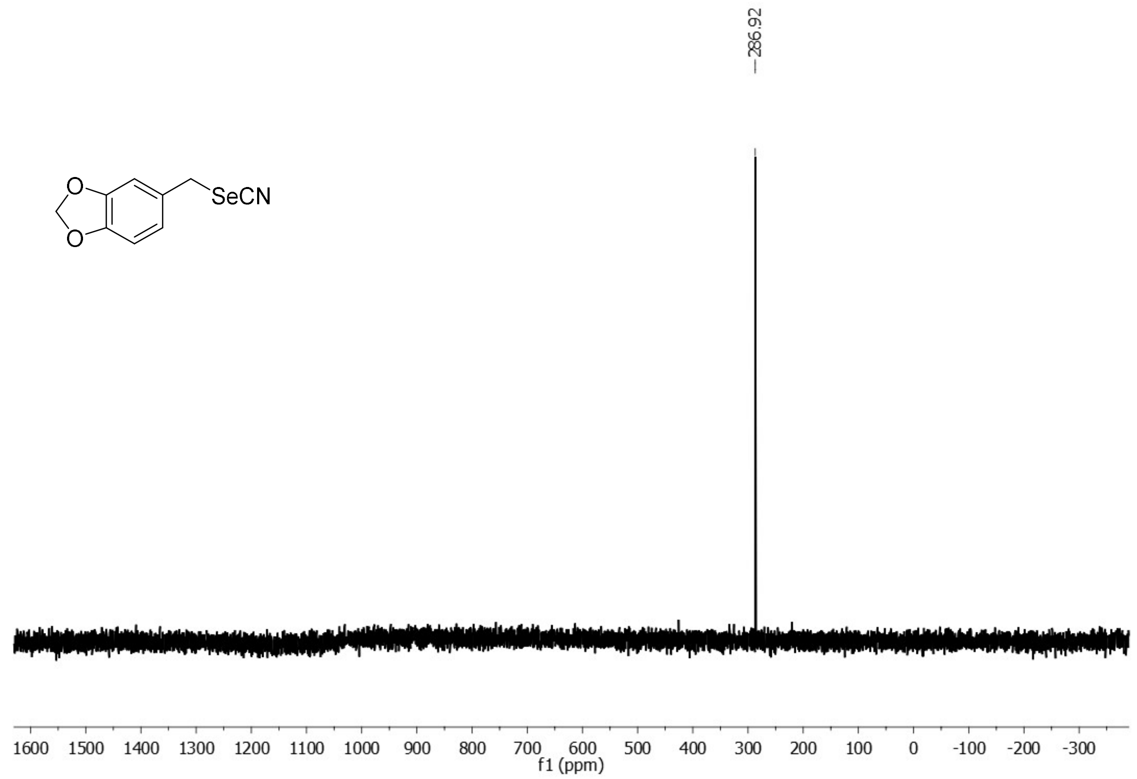

Figure S12. ^{77}Se-NMR spectrum of compound **2a**.

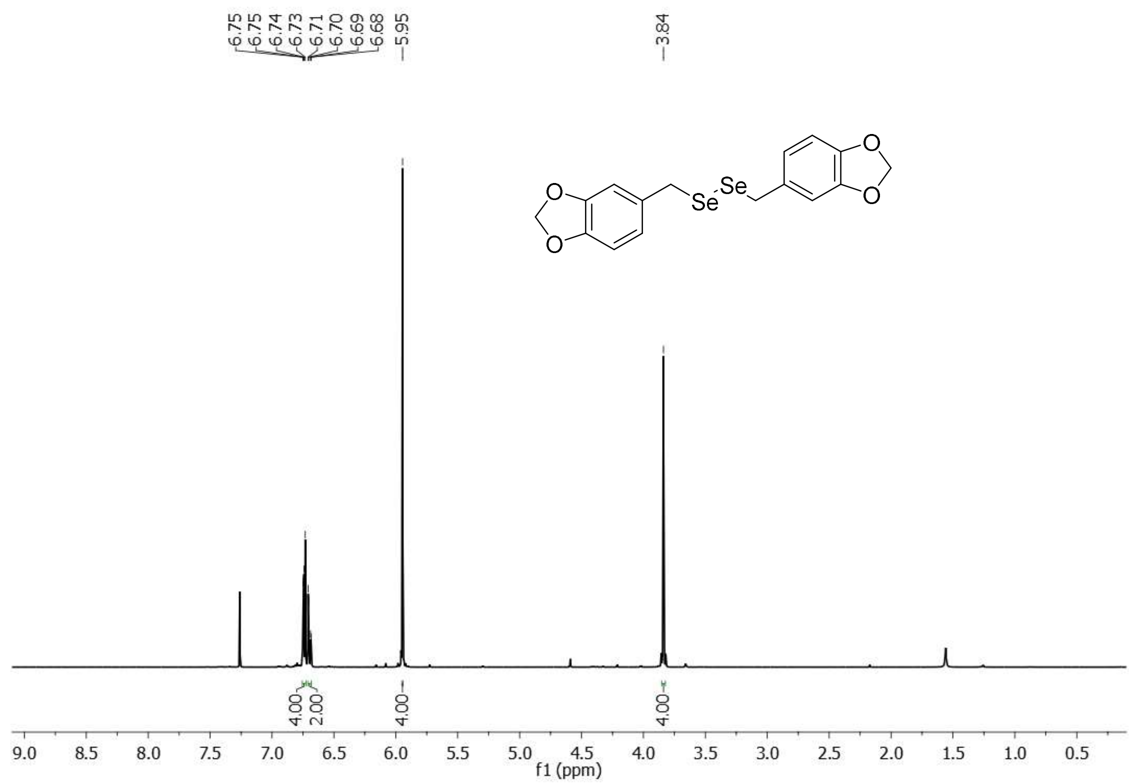

Figure S13. ^{1}H-NMR spectrum of compound **2b**.

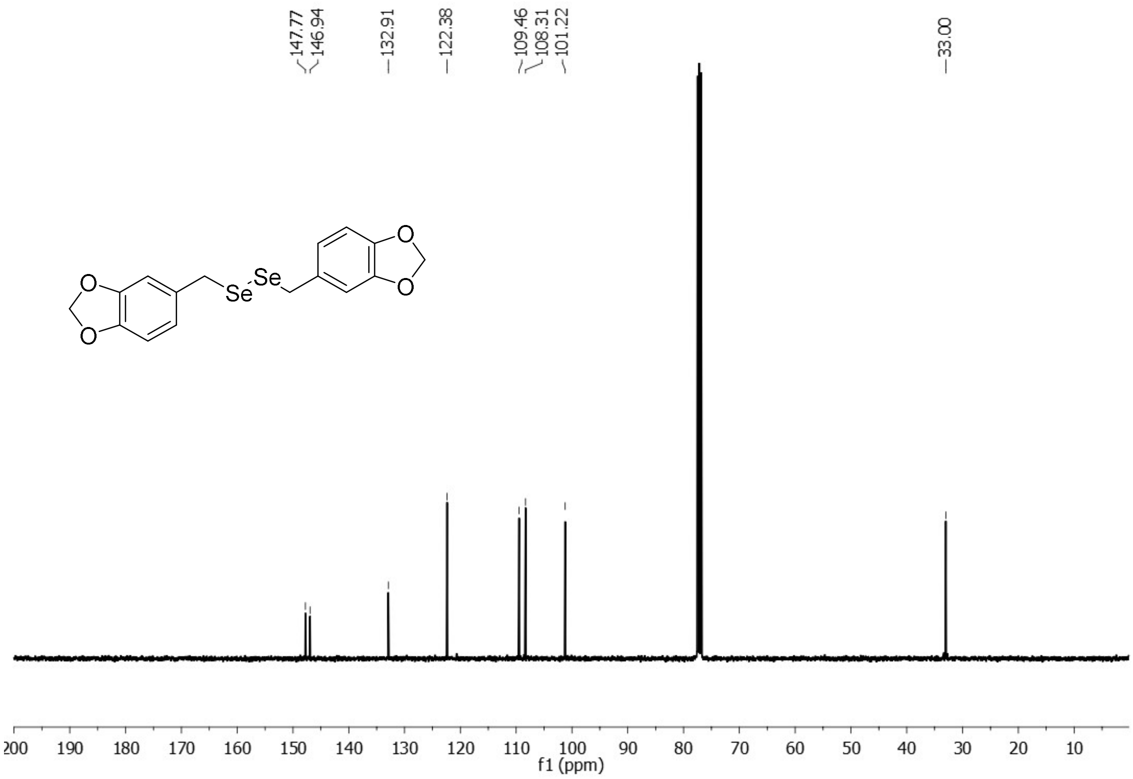

Figure S14. ^{13}C-NMR spectrum of compound **2b**.

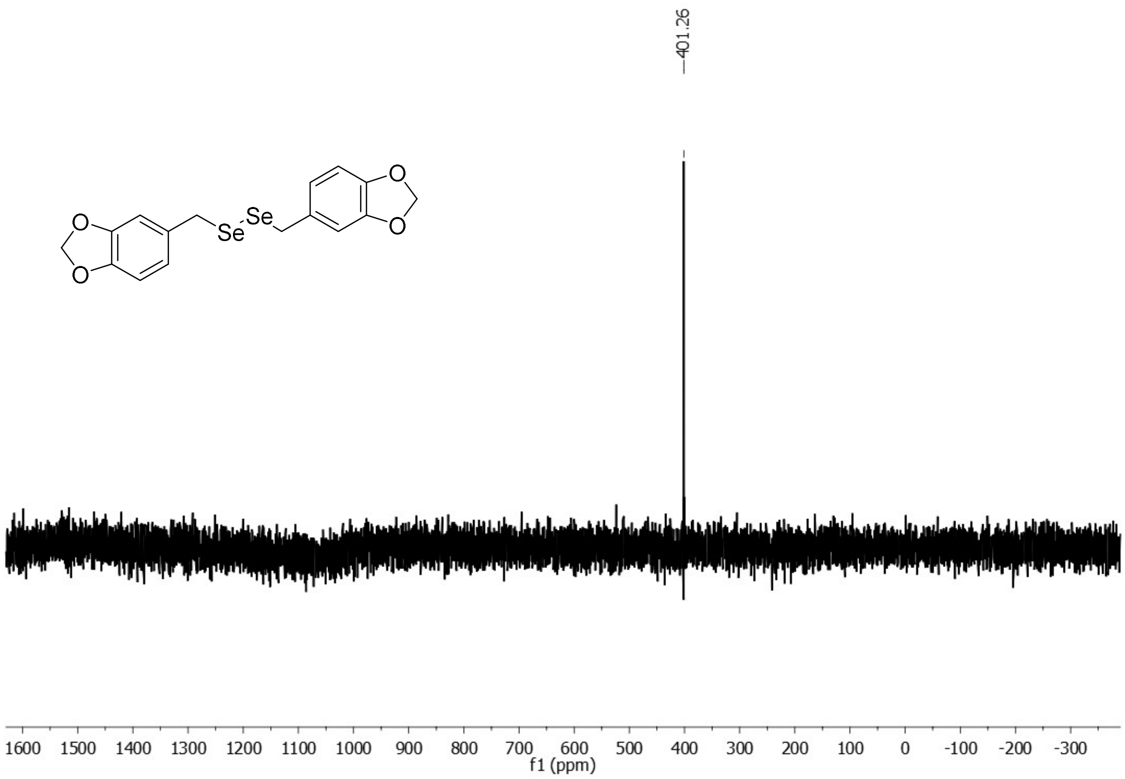

Figure S15. ^{77}Se-NMR spectrum of compound **2b**.

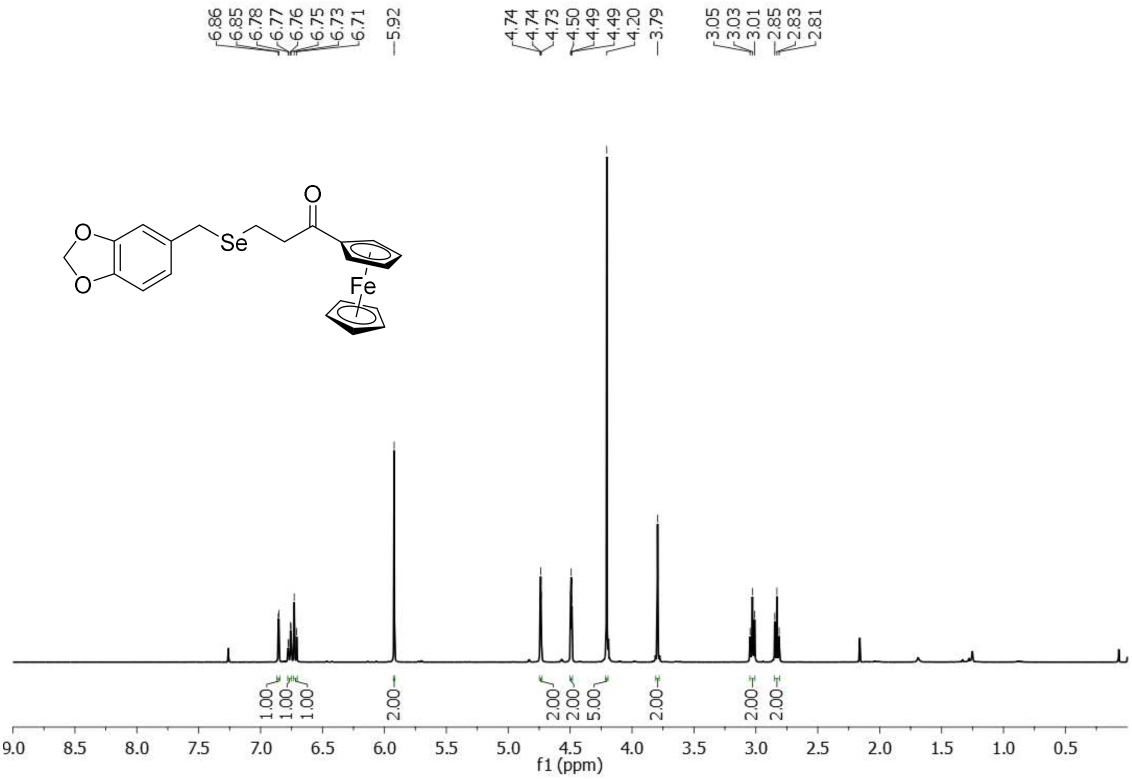

Figure S16. ^{1}H-NMR spectrum of compound **2c**.

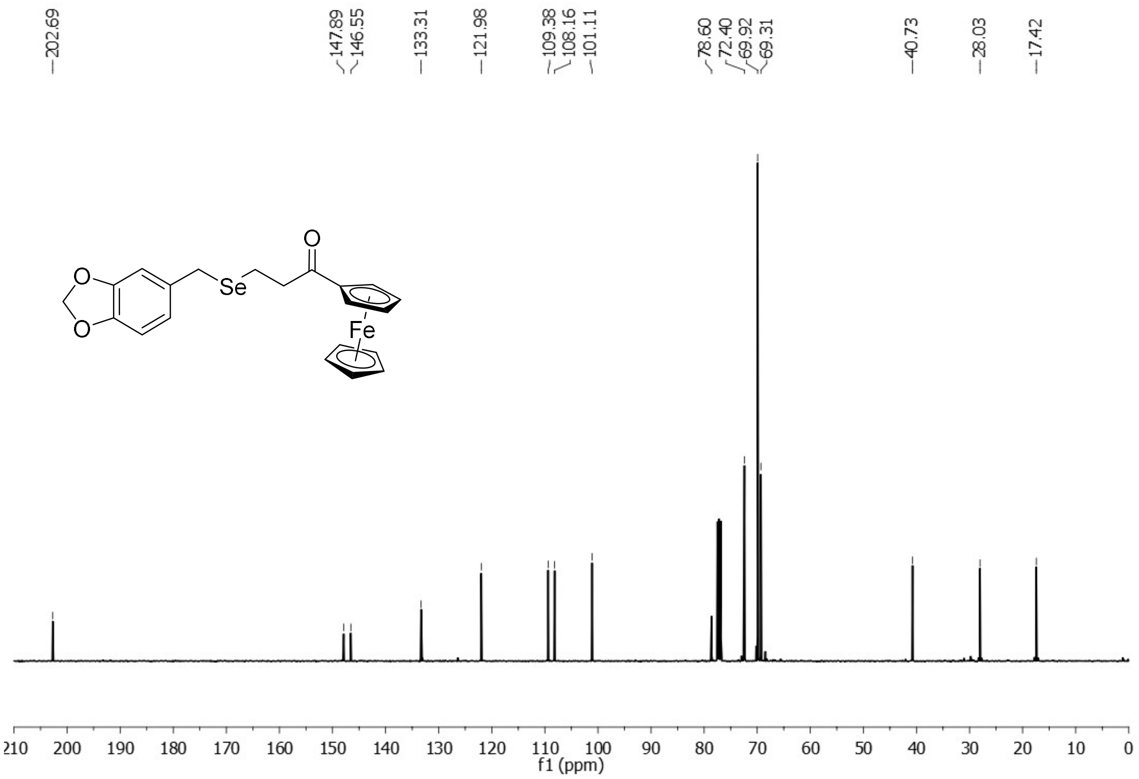

Figure S17. ^{13}C-NMR spectrum of compound **2c**.

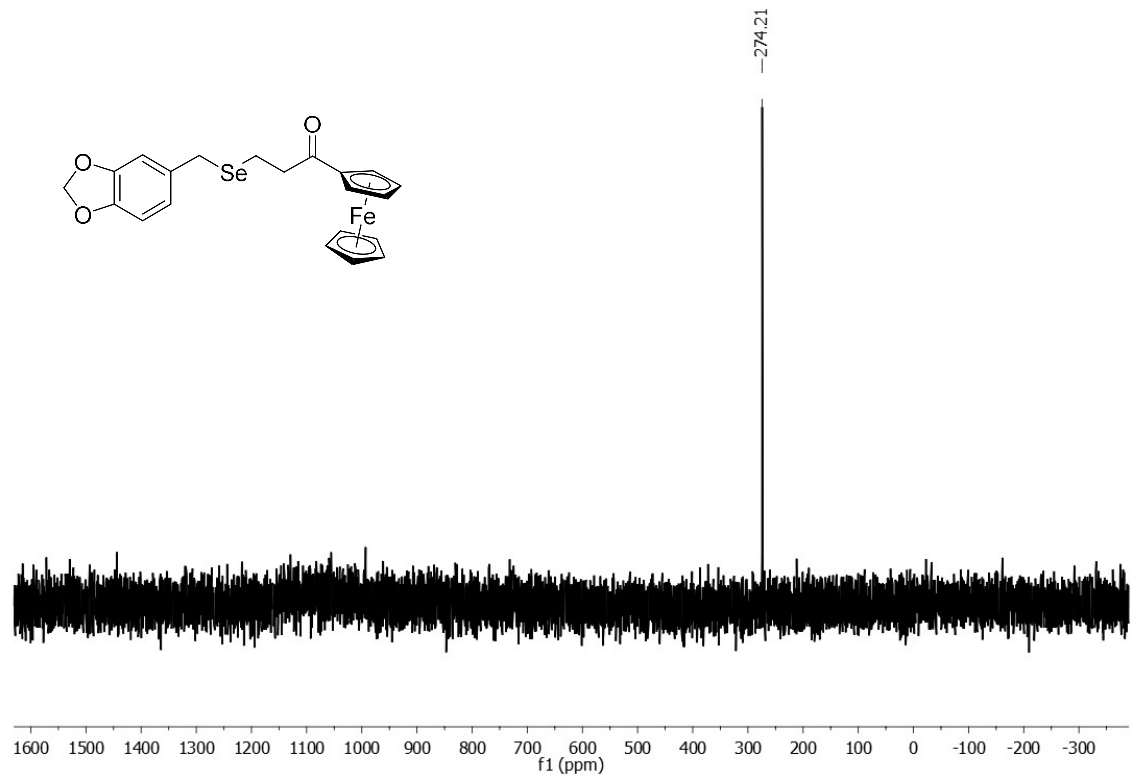

Figure S18. ^{77}Se-NMR spectrum of compound **2c**.
